# Gestational Diabetes Mellitus: A Harbinger of the Vicious Cycle of Diabetes

**DOI:** 10.3390/ijms21145003

**Published:** 2020-07-15

**Authors:** Emilyn U. Alejandro, Therriz P. Mamerto, Grace Chung, Adrian Villavieja, Nawirah Lumna Gaus, Elizabeth Morgan, Maria Ruth B. Pineda-Cortel

**Affiliations:** 1 Department of Integrative Biology and Physiology, Medical School, University of Minnesota, Minneapolis, MN 55455, USA; chung491@umn.edu; 2 Research Center for the Natural and Applied Sciences, University of Santo Tomas, Manila 1015, Philippines; tpmamerto@ust.edu.ph (T.P.M.); adrian.e.villavieja@gmail.com (A.V.); 3 The Graduate School, University of Santo Tomas, Manila 1015, Philippines; gausnawirahl@gmail.com; 4 Baystate Medical Center, Baystate Health, Springfield, MA 01199, USA; lizzmorg@gmail.com; 5 Department of Medical Technology, Faculty of Pharmacy, University of Santo Tomas, Manila 1015, Philippines

**Keywords:** gestational diabetes mellitus and diabetes, polycystic ovary syndrome, obesity, fetal programming, type 2 diabetes, hyperglycemia, glycemic control, insulin, pregnancy complications, diabetes animal models, placenta

## Abstract

Gestational diabetes mellitus (GDM), characterized by a transitory form of diabetes induced by insulin resistance and pancreatic β-cell dysfunction during pregnancy, has been identified as one of the major obstacles in achieving improved maternal and child health. Approximately 9–25% of pregnancies worldwide are impacted by the acute, long-term, and transgenerational health complications of this disease. Here, we discuss how GDM affects longstanding maternal and neonatal outcomes, as well as health risks that likely persist into future generations. In addition to the current challenges in the management and diagnosis of and the complications associated with GDM, we discuss current preclinical models of GDM to better understand the underlying pathophysiology of the disease and the timely need to increase our scientific toolbox to identify strategies to prevent and treat GDM, thereby advancing clinical care.

## 1. Introduction

Improving maternal health and reducing childhood mortality are two of the United Nation’s eight Millennium Development Goals (MDGs). They represent a unique and daunting challenge for healthcare providers worldwide [1,2]. The MDGs are supported by organizations such as the International Federation of Gynecology and Obstetrics (FIGO), which focuses its efforts on the reduction of non-communicable maternal diseases (NCDs) and exposures in pregnancy to improve the future health of women and their offspring. Specifically, FIGO is targeting hyperglycemia, obesity, hypertension, and poor nutrition in pregnancy to reduce the development of diseases later in life, such as obesity and type 2 diabetes mellitus (T2D). This is important, as the goals of early intervention are not only to improve future maternal health but also to reduce disease prevalence in subsequent generations [2].

Pregnancy is often described as a “window” to future health [3], since the physiologic changes that occur during this time act as a natural “stress test” for the body. Many women seek medical care during their pregnancy, which makes this an opportune time for preventive healthcare guidance. In recent years, there has also been a growing realization that the intrauterine environment (e.g., the maternal nutritional status) influences the health of offspring throughout their lifespan [4,5]. The emerging field of the developmental origins of health and disease (DOHAD) posits that intrauterine and early infant environments have a permanent conditioning or programming effect on the body’s metabolism and health later in life.

One common physiological change that can occur during pregnancy is the development of glucose intolerance causing hyperglycemia. This is referred to as gestational diabetes mellitus (GDM). The pathophysiology of GDM is not fully understood but has been linked to hormonal imbalances affecting insulin sensitivity and pancreatic β-cell dysfunction [6]. It is estimated that one in every six pregnancies worldwide is associated with hyperglycemia, 84% of which are classified as GDM [2]. GDM is an important determinant of the development of T2D in both mothers and their offsprings, and thus, achieving glycemic control during pregnancy may provide a window of opportunity to prevent and lower the burden of T2D in many generations.

The goal of this review is to elaborate on how peripartum nutrition affects longstanding maternal and neonatal outcomes, as well as health risks that likely persist into future generations. We aim to summarize current knowledge and data on GDM, with a specific emphasis on screening, diagnosis, and peripartum complications and preclinical models of the disease. Understanding risk factors and improving diagnosis will allow earlier identification and intervention, with the goal of preventing future complications.

## 2. Gestational Diabetes Mellitus

GDM, defined as glucose intolerance with onset or first recognition during pregnancy [7], is a common antepartum condition impacting about 9–25% of pregnancies worldwide [8,9], with rates fluctuating depending on study populations and diagnostic criteria. GDM is characterized by impaired glucose tolerance as a result of maternal pancreatic β-cell dysfunction, resulting in the insufficiency of insulin to regulate glucose homeostasis during pregnancy [10].

Insulin, an anabolic hormone released by the β-cells in the pancreas, modulates glucose homeostasis by stimulating glucose uptake into peripheral tissues, inhibiting glucose production by the liver, and suppressing stored lipid release from the adipose tissue. Insulin resistance is a state in which normal concentrations of insulin fail to achieve an appropriate biological response downstream of the insulin receptor. As a result, the β-cells have to release more insulin than usual to regulate maternal blood glucose levels. In a healthy pregnancy, a condition of progressive insulin resistance occurs in the mother, triggered by placental hormones to ensure the fetus receives adequate nutrients for healthy growth and development. In order to maintain glucose homeostasis despite insulin resistance, the maternal β-cells compensate by increasing total cell number, insulin synthesis, and insulin secretion [11]. However, when the maternal β-cells are unable to adapt to the metabolic changes accompanying pregnancy, hyperglycemia of GDM occurs.

## 3. Risk Factors for GDM

### 3.1. Modifiable Risk Factors

#### 3.1.1. Overweight, Obesity, and Pre-Pregnancy Body Mass Index (BMI)

During pregnancy, maternal dyslipidemia is a physiological response that provides fuel and nutrients for both the placenta and the developing fetus [12]. It is not unusual to observe weight gain in pregnancy, marked by deposition and hypertrophy of adipocytes in the maternal adipose tissue [13]. While pregnant mothers are expected to gain about 30% of their gestational weight in body fat [14], overweight and obesity are the most associated risk factors for GDM [15]. Pre-pregnancy BMI alone is an important risk factor for GDM [16]. The World Health Organization (WHO) defines overweight and obesity as an abnormal or excessive fat accumulation based on BMI > 25 kg/m^2^ or > 30 kg/m^2^, respectively. Given the current epidemic of obesity and the increased rate of obesity in child-bearing women, it is estimated that there are 38.9 million overweight and 14.6 million obese pregnant women worldwide [17]. For people who are overweight and obese, increased lipid production leads to the accumulation of lipids, mainly triglycerides, in the adipose tissue and other organs, such as the liver. Hepatic insulin resistance is increased in obesity and is further exacerbated by pregnancy, thus increasing the risk of developing GDM [18]. Moreover, being overweight or obese during pregnancy can increase the risk of adverse consequences such as metabolic disorders: hypertension [19], premature delivery [20], and stillbirth [21,22], as well as others [23]. For these reasons, obstetricians regularly assess the BMI and gestational weight gain of pregnant women [24] in order to prevent complications for both mother and child. The current prevention strategies to manage body weight during pregnancy include nutritional therapy and improving dietary and lifestyle habits [25,26,27].

#### 3.1.2. Metabolic Syndrome and Nutritional Diet

The term metabolic syndrome was first used by the National Cholesterol Education Program Adult Treatment Panel III to describe the clustering occurrence of metabolic disorders such as obesity, dyslipidemia, hypertension, and abnormal glucose metabolism under one common condition. Metabolic syndrome when accompanied by a western-style diet high in sweets, fats, and processed foods, is known to increase the occurrence of GDM [28,29,30,31,32]. In addition, vitamin D deficiency and high dietary acid load may also contribute to the risk of GDM [33].

Notably, the first line of prevention or treatment for metabolic syndrome is nutritional diet therapy, which has been shown to be beneficial in maintaining glucose control and physiologic health [34,35,36]. Nutritional therapies, such as high-fiber and low-glycemic-index diets, have been shown to improve insulin sensitivity and glucose tolerance, which may reduce the risk for GDM [37,38,39,40,41,42]. Recent literature has also shown the benefits of probiotic therapy in improving blood glucose levels by targeting the gut microbiota [43,44,45]. Finally, anti-hyperglycemic medications, such as insulin therapy and metformin, can be introduced when lifestyle modifications alone are not sufficient to achieve glycemic control [46].

#### 3.1.3. Polycystic Ovary Syndrome (PCOS)

GDM and polycystic ovary syndrome (PCOS) are the most common endocrine disorders in women of reproductive age. PCOS is a heterogeneous endocrine and metabolic disorder characterized by chronic oligomenorrhea [47], hyperandrogenism [48], and insulin resistance [49]. Like GDM, PCOS is associated with insulin resistance and obesity [50]. While the risk for GDM increases for women with PCOS in the presence of other comorbidities, such as obesity and increased maternal age, PCOS per se is not an independent risk factor for GDM [51,52]. In a prevalence study in California, data showed that pregnant women with PCOS have more than a twofold risk for GDM compared with women without PCOS or related symptoms [53]. Indeed, the prevalence of PCOS is higher in women with GDM than in non-diabetic women [51]. PCOS is often associated with metabolic syndrome, and common prevention strategies include lifestyle modification [54,55] and pharmacological treatments [56].

#### 3.1.4. Pre-Eclampsia

Pre-eclampsia is a common hypertensive disorder that occurs in 2–8% of all pregnancies worldwide [57,58]. Like GDM, pre-eclampsia is linked to glucose intolerance, hyperglycemia, and obesity [59]. Hyperglycemia is known to increase the risk of pre-eclampsia [60,61,62]. In subsequent pregnancies, pre-eclampsia is an independent risk factor for GDM [61].

#### 3.1.5. Additional Modifiable Risk Factors for GDM

A few studies have determined that prolonged exposure to environmental psychological stress is related to maternal hyperglycemia during pregnancy, which may increase the risk of GDM [63]. Similarly, the use of antidepressant and psychotropic medications, smoking, and poor sleep hygiene have also been shown to be risk factors for GDM [64,65,66].

### 3.2. Non-Modifiable Risk Factors

#### 3.2.1. Maternal Age

Maternal age is a common risk factor for GDM. Studies have shown that maternal age above 25–30 years increases the risk of developing GDM [67,68,69,70]. In a meta-analysis study investigating the relationship between maternal age and the risk of GDM, authors identified a linear relationship between the risk for GDM and increasing maternal age. It was also indicated that for every successive year after the age of 18, the risk for GDM increases by 7.90%, 12.74%, and 6.52% in the general, Asian, and Europid populations, respectively [71].

#### 3.2.2. Gravidity and Parity

Increasing gravidity, defined as the number of times that a woman has been pregnant, and parity, i.e., the number of times that she has given birth, may represent an additional risk for GDM [69]. This elevated risk of developing GDM has been linked to parities as low as two [67,72]. The effect of gravidity on the risk of GDM has been associated with increasing age as well, since an increased number of pregnancies is observed in women with advanced maternal age [73].

#### 3.2.3. Ethnicity

Several studies have linked GDM development with ethnicity. Increased risk of GDM is seen in multiple ethnic and racial groups, including Hispanic, African American, and Asian women [73,74]. Women of Korean, Chinese, and Filipino descent are more than twice as likely to develop GDM as Caucasian or African American women [75]. Although the mechanisms remain unclear, possible explanations can arise from health predisposition, lifestyle, cultural factors, and socioeconomic stressors [71,73]. In regards to T2D studies, South Asians have been reported to have reduced fat metabolism, muscle fitness, insulin sensitivity, and insulin secretion, all of which support a higher tendency towards glucose intolerance [76,77]. Taking ethnicity as a risk factor for GDM, it is therefore important for health providers to recognize that certain ethnic groups may benefit from special preventive and culturally sensitive care. Further, structural changes, such as combatting systemic racism and bias are necessary to break down disparities associated with these preventable NCDs.

#### 3.2.4. Genetics and Family History of Hyperglycemia

GDM is a multifactorial disease with both genetic and environmental influences. A family history of diabetes is an important independent risk factor for the development of GDM [78]. There is a strong association between common T2D risk gene polymorphisms and GDM [79]. Five of the most identified genes in GDM are: (1) Transcription factor 7-like 2 [TCFL7L2] [80,81]; (2) Melatonin receptor 1B [MTNR1B] [82,83]; (3) CDK5 regulatory subunit-associated protein 1-like 1 [CDKAL1] [84,85]; (4) Potassium voltage-gated channel, KQT-like subfamily, member 1 [KCNQ1] [86,87]; and (5) Insulin receptor substrate-1 [IRS1] [88,89]. The most recent and comprehensive searches in the genetic and epigenetic etiology of GDM have been reviewed elsewhere [90,91]. These epigenetic modifications may result in maternal and paternal transgenerational inheritance of obesity and glucose intolerance in the offspring [92,93,94]. Moreover, the types of genetic variation and the mechanisms of epigenetics may contribute to genotypic and phenotypic characteristics in different ethnicities [95,96]. For example, studies that explored the relationship between ethnicity and GDM revealed that Asian women have the highest GDM incidence [97,98]. The variability in the association of genetic polymorphisms and risk of GDM has been attributed to ethnicity or population differences; thus, there is a need to perform population-dependent studies on the effect of genetic polymorphisms on the risk of GDM [99,100].

### 3.3. Socioeconomic and Geographic Risk Factors

#### 3.3.1. Climate and Geographical Location

The WHO recognizes that varying climate conditions from extreme winter temperatures to summer temperatures have an impact on human health. There is increasing evidence showing the influence of temperature on physiologic mechanisms such as the regulation of fats and lipids [101,102], energy expenditure, hormonal homeostasis [103], myocardial infarction [104], and mortality [105]. Climate also appears to have an effect on GDM. In regions with seasonal weather changes there is a higher prevalence of GDM than in more temperate regions of the world [106].

#### 3.3.2. Education and Socio-Economic Status

Based on observational studies, many women diagnosed with GDM are unaware of the risk factors and complications of their diagnosis [107,108,109]. A study in Finland reported that there was an inverse relationship between socioeconomic status and GDM [110]. This is supported by findings that patients who are uninsured and underinsured receive less preventive healthcare, with some receiving little or no healthcare at all [111,112]. To improve the efficacy of GDM treatment, promoting health education combined with government support to patients is an important component of prenatal care [113]. Several strategies shown to be beneficial in promoting awareness of GDM and improving pregnancy outcomes include the use of web-based education [97] and/or of individual or group educational sessions with a healthcare provider or a dietician [114,115,116].

### 3.4. Screening and Diagnosis

#### 3.4.1. Laboratory Evaluation

To fully address the increasing prevalence of GDM worldwide, routine screening for GDM during prenatal care is necessary. However, only few countries routinely test pregnant women universally. In the United States, the American College of Obstetricians and Gynecologists (ACOG) recommends all pregnant women be tested in the mid-trimester (between 24 and 28 weeks of gestation) [117]. In some countries, testing is completed based on the risk assessment done by the Obstetrician–Gynecologist. Risk is categorized by doctors as low, average, or very high. Low-risk individuals are those whose age is below 25 years, with normal pre-pregnancy weight, members of an ethnic group with a low prevalence of diabetes, no known diabetes in first-degree relatives, no history of abnormal glucose tolerance, and no history of poor obstetric outcomes.

Although there is no international, standardized consensus on the screening test to be used, the oral glucose tolerance test (OGTT) is commonly utilized [118,119]. There are two current approaches for testing GDM. In the one-step approach, a single 75 g OGTT is conducted, while in the two-step approach, an initial screening using a 50 g oral glucose challenge test (OGCT) is performed; if the results of this test show a glucose value equal or greater than 7.7 mmol/L (130–140 mg/dL) [117], the pregnant woman analyzed should undergo a confirmatory 100 g OGTT several days after the OGCT [120,121,122]. Table 1 below shows the comparison between the one-step approach and the two-step approach.

Numerous studies have evaluated these screening modalities. One study compared the prevalence and pregnancy outcomes of Thai GDM patients screened using the one-step, 75 g OGTT and the two-step approach. The results showed a very high prevalence of GDM when the one-step approach was utilized; however, there was no clear evidence of better performance of this test. This higher prevalence seen using the one-step approach was associated with the use of a lower threshold for diagnosis, which thus led to an increased sensitivity [123]. A higher sensitivity may result in an increased number of false positives and overdiagnosis and treatment, with increased risk of unnecessary intervention, higher costs, and decreased maternal satisfaction with testing and prenatal care [125]. The two testing approaches present advantages and disadvantages. While the one-step approach has a lower economic burden and a lower threshold, allowing for the earlier diagnosis of milder disease and possible early prevention of complications in the mother and baby [124,126], the two-step approach is less convenient economically and requires more time for diagnosis, though it still provides diagnostic efficacy [127].

#### 3.4.2. Criteria for GDM Diagnosis

The selection criteria for the diagnosis of GDM are controversial because of issues in determining the standard reference intervals for GDM diagnosis. The standardization of global criteria is a challenge because GDM is influenced by genetics, ethnicity, and socioeconomic and societal factors. The following health professional and study groups have proposed their own diagnostic criteria: American Diabetes Association (ADA) (187); WHO; International Association of Diabetes and Pregnancy Study Group (IADPSG) (184,186); Australasian Diabetes in Pregnancy Society (ADIPS) [128]; Diabetes Canada [129]; German Association for Gynecology and Obstetrics (DGGG) [130]; Istituto Superiore di Sanità (ISS) [131]; Hyperglycemia and Adverse Pregnancy Outcomes (HAPO) Study Cooperative Research Group [128,132,133,134,135]; and The Swiss Society for Endocrinology and Diabetes [136]. Table 2 below shows the most common diagnostic criteria utilized for GDM diagnosis. Varying criteria for diagnosis must be considered, as they may affect the stated prevalence and outcomes of GDM. 

### 3.5. Complications

GDM is characterized by hyperglycemia diagnosed during pregnancy, caused by or compounded with underlying mechanisms such as genetic predisposition, insulin resistance, and chronic inflammation. Although the condition is usually transient, it is a risk factor for the development of T2D later in life and may also lead to long-term adverse effects in both mother and offspring. This session enumerates the possible metabolic and physical changes resulting from GDM development.

#### 3.5.1. Maternal Complications

In GDM, hyperglycemia may damage endothelial cells, which can result in vascular dysfunction [138] associated with hypertension [139]. Because of this, it is has been suggested that GDM increases the incidence of hypertension during pregnancy and the postpartum period [140]. Both diabetes and hypertension are risk factors for the development of pre-eclampsia, a disorder which affects between 3% and 5% of pregnancies worldwide and is characterized by high blood pressure and proteinuria [141,142].

Although hyperglycemia during pregnancy usually resolves after delivery, prolonged insulin resistance and β-cell dysfunction can also be observed in GDM patients, persisting beyond pregnancy [143]. Because of this, women with previously diagnosed GDM have an increased risk of developing T2D later in life [144,145,146,147,148], with a risk high as 50% [149]. Also, women who have developed GDM in previous pregnancies may experience a recurrence of GDM in subsequent pregnancies [150,151]. For this reason, all women who carry a diagnosis of GDM should have a 2 h glucose tolerance test at their 6-week post-partum visit [117].

#### 3.5.2. Fetal Complications

The developing fetus has a limited ability to produce glucose; therefore, it derives most of its glucose from maternal blood. Maternal glucose crosses the placenta, while maternal insulin does not. As a result, according to the modified Pedersen’s hypothesis, if maternal glucose levels are high and uncontrolled, the excess glucose transported through the placenta induces increased fetal insulin production regardless of glucose stimulation [152]. This is supported by the observed increase in the expression of glucose transport proteins (GLUTs) in the placenta in pregnancies affected by insulin-dependent diabetes mellitus [153]. Insulin can also stimulate mTOR, a potent regulator of cell growth [154]. An increase of placental mTOR activity due to increased maternal insulin results in increased cell proliferation and nutrient transport to the fetus via System A and System L amino acid transporters in the placenta [155,156]. Hart et al. reviewed the role of mTOR as a nutrient sensor in fetal growth [157].

Because of the aforementioned factors seen in GDM, maternal hyperglycemia and hyperinsulinemia can lead to similar changes in the fetus [158,159], which can contribute to neonatal adiposity [160]. Excess nutrient storage results in an increase in neonatal size at birth, or macrosomia. Between 15% and 45% of GDM pregnancies result in macrosomic infants [152], with the bulk of adiposity concentrated around the fetal abdomen and shoulders, increasing the risk for shoulder dystocia and birth trauma [161]. The presence of GDM, coupled with other risk factors like hypertension and obesity during pregnancy, may also lead to preterm labor and birth [162], a prevalence that reaches about 10.6% worldwide [163].

#### 3.5.3. Neonatal Complications

Neonatal complications include possible asphyxia, hypoglycemia, kernicterus and jaundice, bacterial infections, neonatal respiratory distress syndrome (NRDS), and birth trauma, including shoulder dystocia and injury to the brachial plexus [152]. Neonatal hypoglycemia occurs as a result of the abrupt cessation of the maternal source of glucose at birth [164]. This is exacerbated by fetal hyperinsulinemia due to GDM and requires extensive treatment and care if the hypoglycemia persists [165].

#### 3.5.4. Childhood and Adulthood Complications

The association between GDM and hyperglycemia in the offspring is well established. In the United States, the study of the Pima Indians provided initial evidence that maternal hyperglycemia could lead to adult disease in the offspring. Several epidemiological studies demonstrated the Pima Indian population as having the highest prevalence of T2D among children and adults [166]. Indeed, the offspring of diabetic mothers are more often prone to obesity, hypertension, and dyslipidemia later in life [167]. The Hyperglycemia and Adverse Pregnancy Outcome (HAPO) study in 10 countries revealed that maternal hyperglycemia during pregnancy was significantly associated with an increase in hyperglycemia and insulin resistance in the offspring in adulthood [168]. Markers for insulin resistance, like HOMA-IR, BMI, waist circumference, and triglyceride levels, were also at higher levels in GDM offspring than in those born to normoglycemic mothers [169,170]. Presumably, the development of insulin resistance increases the risk of the offspring to develop diabetes, with approximately 20% of GDM offspring developing T2D and prediabetes by age 22 [171,172].

The increased development of obesity observed in the offspring of mothers with GDM is also associated with an increased risk of metabolic disorders including cardiovascular diseases and insulin resistance [173]. Children born to GDM mothers were observed to have significantly higher blood pressure and adiposity, along with hyperglycemia and BMI [174,175]. As a result of increased cardiovascular risk, GDM offspring are more likely to develop cardiac arrhythmias and be hospitalized for cardiovascular diseases (CVDs) [176]. GDM offspring are also 29% more likely to develop early-onset CVDs such as heart failure, hypertensive disease, deep vein thrombosis, and pulmonary embolism [177]. All of these studies point to the influence of the environment in utero in the programming of metabolic disease in the offspring. This is an important factor to consider, because overnutrition, physical inactivity, and/or genetic factors alone are not sufficient to explain the current epidemic increases in T2D and obesity. Monterio et al. reviewed the mechanisms of fetal programming in GDM elsewhere [178].

## 4. Preclinical Models of GDM

To stop the vicious cycle of diabetes, we need a greater understanding of the pathophysiology of GDM and the mechanisms of fetal programming induced by GDM. As previously mentioned, this is an essential endeavor, because GDM confers short- and long-term health risks for the mother and the fetus, with potential long-term health consequences in childhood and adulthood [179]. However, establishing causality is difficult in human and epidemiological studies, which are often complicated by confounding multiple factors. Therefore, experimental animal models are critical to study the underlying mechanisms and pathophysiology of GDM. Methods for generating animal models of GDM are diverse and include the surgical removal of all or part of the pancreas, the use of pharmacological agents, diet-induced strategies, and genetic models [180].

### 4.1. Surgical Models

Surgical models include partial or total pancreatectomy, directly reducing the availability of pancreatic β-cells and dramatically impairing glucose homeostasis [180]. One study performed pancreatectomy in a rat model to reduce the pancreatic mass by 95%, resulting in uterine dysfunction in pregnant rats with mild GDM [181]. While pancreatectomy was successful in inducing GDM, it was performed prior to pregnancy, which does not accurately reflect the development of human GDM. Pancreatectomy has also been shown to induce hyperglycemia and diabetes in healthy baboons; however, such models are rarely used in the context of pregnancy [182]. While the surgical removal of the pancreas may induce maternal diabetes during pregnancy, it is an invasive and nonspecific procedure, as it removes both the endocrine and the exocrine tissues of the pancreas [180]. This may result in potential effects not related to GDM.

### 4.2. Pharmacological Models

Pharmacological agents, including streptozotocin (STZ) and alloxan, have been used to selectively destroy the pancreatic β-cells and impair β-cell function [183,184]. Chemical agents offer a relatively easy way to generate maternal hyperglycemia and diabetes; however, there are inconsistencies in the effects of chemical agents, depending on drug delivery method, dosing, species, age, diet, and time of gestation at which the drug is administered. While rodents are more commonly used as models for chemical-induced diabetes studies, STZ has been used in nonhuman primates to study the effects of maternal diabetes on the offspring [185]. In female rhesus monkeys, STZ treatment induced hyperglycemia and glucose intolerance [185,186]. The treated animals were also found to have larger placentas and neonates, as well as a higher incidence of stillbirth [186]. While both surgical and chemical animal models have been used to reproduce GDM, neither are able to accurately simulate the conditions of human GDM. Pancreatectomy and the use of STZ and alloxan permanently remove the endocrine function of the pancreas, reducing insulin and resulting in a permanent state of diabetes. This is unlike GDM in humans, usually a transitory disease that develops as a consequence of maternal insulin resistance, compounded by increasing amounts of human placental lactogen throughout pregnancy and the inability of the maternal pancreatic β-cells to adapt [180]. It is also important to acknowledge that there can be fundamental similarities and important differences between rodents and humans regarding islet biology. For example, there is still a debate on β-cell compensation during pregnancy in rodent models vs. humans [187], because limited human autopsy studies do not consistently support the mechanisms observed in rodents.

### 4.3. Diet-Induced Models

Diet-induced models of GDM include high-fat feeding in animal models to induce insulin resistance and diabetes. A study using a high-fat diet (HFD) demonstrated that in the non-pregnant state. female rats, while obese, displayed normal glucose clearance. After successful mating with control males, pregnant females on HFD displayed hyperglycemia and glucose intolerance [188]. In another study, pregnant female rats were administered continuous glucose infusions during the last week of gestation, inducing hyperglycemia and hyperinsulinemia [189]. The offspring born to these females had phenotypes resembling those of children born to mothers with GDM. While a GDM animal model involving obesity may represent actual risk factors for human GDM, these models do not consider the genetic and social factors contributing to the development of the disease.

### 4.4. Genetic Models

Genetic models have been used to induce GDM in animals. The db/db mouse model of leptin deficiency is currently the most widely used model of T2D. Normally, db/+ female animals present a normal glucose homeostasis phenotype; however, during pregnancy, they develop spontaneous GDM, and pups display characteristics similar to those of infants of GDM mothers [190]. For example, the offspring of db/+ dams with GDM exhibited obesity and insulin resistance in the liver. Another model that has been described is the prolactin receptor deficient (PrlR^−/−^) mouse. While PrlR^−/−^ females were unable to carry a pregnancy to full term, Prl^+/−^ dams exhibited hyperglycemia and failure to increase β-cell mass and proliferation during pregnancy, a necessary event to maintain euglycemia [191]. In the non-pregnant state, these female mice presented with euglycemia and decreased β-cell mass. Other genetic models that have investigated transcription factors and different key signaling pathways to induce GDM are discussed elsewhere [180,192,193]. Genetic models provide an opportunity to study the underlying mechanisms involved in the pathogenesis of GDM. Unfortunately, conclusions may be limited, as they are often based on single gene mutations, which do not accurately mimic the polygenetic and environmental factors contributing to human GDM. 

### 4.5. Fetal Programming Models

Several novel models of GDM have been generated by using the first-generation offspring (F1) of dams (F0) from various intrauterine programming procedures. In a study using a rat model, STZ, a toxin that promotes pancreatic β-cell demise, was administered to F0 generation female pups to induce diabetes [194]. After successful mating with nondiabetic males, the female F1 pups were used to study GDM during pregnancy. They found that non-pregnant F1 pups were euglycemic but developed hyperglycemia and hyperinsulinemia during pregnancy. This suggests that exposure to a diabetic intrauterine environment may set up female offspring to develop GDM during pregnancy. In another study, a uteroplacental insufficiency rat model was used to generate intrauterine growth-restricted (IUGR) offspring [195]. After successful breeding of the IUGR female offspring to normal males, pregnant females demonstrated glucose impairment and insulin resistance. The offspring of these female rats exhibited increased body weight, insulin resistance, and impaired glucose clearance, eventually developing diabetes. In another mouse model, researchers used the F1 offspring born to dams fed a low-protein (LP) diet during gestation and lactation. The F1 females develop glucose intolerance and reduced β-cell proliferation during pregnancy [196]. These models demonstrated that exposure to GDM or glucose intolerance during gestation increases the susceptibility of the offspring (F1) to developing GDM.

Animal models have also been used to study the consequences of GDM on the health of the offspring. Offspring born to mothers with GDM are at increased risk for obesity, glucose intolerance, and diabetes [179]. In the previously mentioned study by Gauguier et al., in which GDM was induced in female rats by continuous glucose infusion administered during the last week of pregnancy [189,197], female offspring demonstrated glucose intolerance and impaired insulin secretion. When these female offspring were mated with control males, the newborn offspring presented with hyperglycemia, hyperinsulinemia, and increased body weight, which persisted into adulthood. These studies suggest that intrauterine exposure to maternal diabetes may affect the health of more than one generation. In the intrauterine programming model of maternal gestational diabetes in rats by Boloker et al., the offspring developed glucose intolerance and impaired insulin secretion, which worsened with age [195]. The effects of maternal high-fat diet on the health of the offspring have also been studied in nonhuman primates. In female Japanese macaques, maternal high-fat diet was shown to increase liver triglycerides and increase the risk of developing nonalcoholic fatty liver disease in the offspring [198,199]. These studies provide evidence that there are lasting effects of maternal gestational diabetes on the health of the offspring.

GDM is a complex disease with genetic, environmental, and epigenetic risk factors. Therefore, it is unlikely that a single animal model will accurately represent human GDM. A realistic model may include animal models with multiple etiologies presented by GDM, which will likely include both genetic and environmental factors. Furthermore, while larger animals and nonhuman primates may be more similar to humans physiologically, studies using these animal models remain more limited compared to those on mice and rat models, due to feasibility and costs.

Nevertheless, animal models have advanced the understanding of maternal gestational diabetes beyond clinical observation alone. Furthermore, they may provide a valuable means to study intervention strategies targeting GDM in pregnant women.

## 5. Concluding Remarks

**Figure 1:**
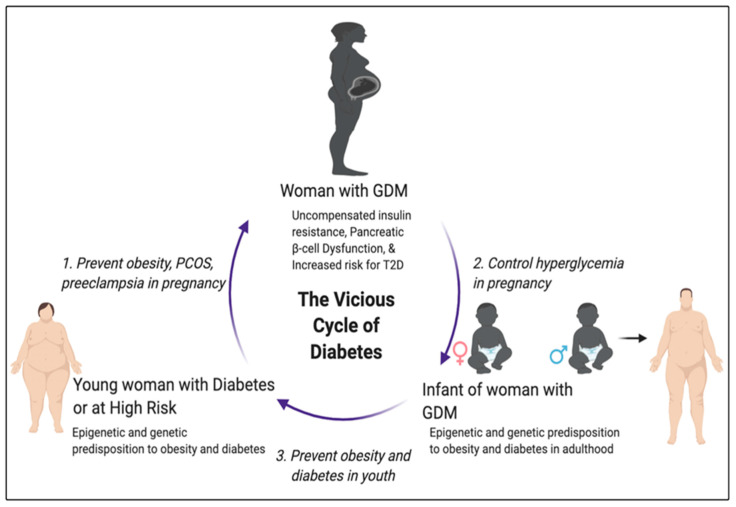
Gestational diabetes mellitus (GDM) is a harbinger to the vicious cycle of transgenerational obesity and diabetes. The general pathology of GDM is shown, including the three critical windows of opportunity to break the cycle. PCOS, polycystic ovary syndrome.

The high prevalence of GDM is a major obstacle to achieving improved maternal and child health. While GDM is a transient condition, its sequalae are lifelong, and GDM research should be a public health priority. Studies discussed in this review underscore the gravity of GDM and challenges to its management, including identifying and overcoming risk factors, accurate diagnosis, and treatment of the disease in order to prevent associated complications. We emphasized the health burden and consequences of GDM in both the mother and the baby, as well as in subsequent generations (Figure 1). We highlighted the utility of preclinical models of GDM to help us understand the underlying pathophysiology of the disease and the timely need to increase our scientific toolbox to identify strategies to prevent and treat GDM, thereby advancing clinical care. The prevalence of obesity and diabetes continues to increase worldwide and, until we can put a stop to the vicious cycle of diabetes, will continue to impose great burdens on patients, their families, and society as a whole. As depicted in Figure 1, there is a high need to prioritize preventive healthcare for pregnant women at risk for GDM. There are critical points during this vicious cycle of transgenerational obesity and diabetes which offer opportunities for intervention. We stress here that the continued study of GDM including its risk factors, diagnosis, and management is necessary for its understanding as well as for the prevention of metabolic disease in the offspring and for stopping the vicious cycle of diabetes.

## Figures and Tables

**Table 1:** Advantages and disadvantages of the one-step and two-step approaches of glucose testing [120,121,122,123,124]. GDM, gestational diabetes mellitus, Neonatal Intensive Care Unit (NICU), OGTT, oral glucose tolerance test.

Type of Testing	Advantages	Disadvantages
One-step approach	Simple to follow Better patient adherence Easy diagnosis Cost-effective for high-risk individuals Increased sensitivity Detection of milder GDM, thus less complications like pre-eclampsia, applicable to women of any gestational age, neonatal hypoglycemia, neonatal death, and NICU admission	Poor reproducibility Women need to be in a fasting state
Two-step approach	Fewer false positive results Avoids OGTT in more than 75% of women	Less patient compliance Requires patients to make two visits for testing Missed diagnosis: 75% sensitivity with 84% specificity as compared with the single-step, 100 g OGTT Delay in initiating treatment even for those who tested positive

**Table 2:** Commonly used diagnostic criteria for GDM [118,137]. ADA, IADPSG.

75-g OGTT	Glucose Threshold for Diagnosing GDM in mmol/L (mg/dL)		
**GLUCOSE MEASURE**	ADA **	IADPSG *	WHO *
FASTING	5.1 (92.0)	5.1 (≥92.5)	5.1–6.9 (92.0–125.0)
1 H	10.0 (180.0)	10.0 (≥180.0)	≥10.0 (180.0)
2 H	8.5 (152.0)	8.5 (≥153.0)	8.5–11.0 (153.0–199.0)

Legend: * any one value meeting the threshold leads to a diagnosis of GDM; ** any two values meeting the threshold lead to a diagnosis of GDM.
